# Accelerated Idioventricular Rhythm During Ajmaline Test: a Case Report

**Published:** 2010-10-31

**Authors:** Antonio Sorgente, Yoshinao Yazaki, Lucio Capulzini, Andrea Sarkozy, Carlo de Asmundis, Gian-Battista Chierchia, Mehmet Stephan-Andreas, Pedro Brugada

**Affiliations:** 1 Heart Rhythm Management Center, UZ Brussel-VUB, Laarbeeklaan 101, 1090 Brussels (Belgium); 2 University of L’Aquila, Piazza Salvatore Tommasi 1, 67010 Coppito, L'Aquila (Italy)

**Keywords:** Brugada syndrome, ajmaline test, accelerated idioventricular rhythm

## Abstract

We present an unusual transient pro-arrhythmic effect of ajmaline in a patient with resuscitated cardiac arrest and a left ventricular apical aneurysm. We discuss the clinical presentation and the possible physio-pathological explanation for this new pro-arrhythmic effect linked to administration of intravenous ajmaline.

## Introduction

Brugada syndrome (BrS) is a well-recognized arrhythmogenic disease associated with an increased risk of sudden death related to ventricular tachyarrhythmias or ventricular fibrillation [1]. Sodium channel blockers drugs help clinicians to overcome one of the major characteristics of the disease, the wide variation in the ECG pattern [2]. Among sodium channel blockers, ajmaline has been demonstrated to be the one with highest predictive power [3], possibly because of its kinetics and strength of rate-dependent sodium channel blocking effects.

A potential pro-arrhythmic effect of class I drugs has been reported [4]. Ajmaline challenge has been associated with lethal arrhythmias (polymorphic ventricular tachycardias and ventricular fibrillation), even in patients without BrS [5].

## Case presentation

We present the case of a 29 year-old man admitted to our hospital after a resuscitated cardiac arrest. His family history was negative for sudden cardiac death and channelopathies. His past medical history was unremarkable: the patient was completely asymptomatic and never complained of syncope or palpitations. Moreover, on clinical examination he didn't show any apparent abnormality. Electrocardiographic findings were completely normal. An exercise testing was clinically and electrically negative but rare isolated ventricular ectopic beats were documented during the test and in the recovery phase. For this reason the patient was planned for a 24 hour Holter ECG registration. In the afternoon following the exercise testing, during a small effort, the patient experienced cardiac arrest. In the emergency room, he was found to be in ventricular fibrillation. Four DC shocks and iv adrenaline and cardio-pulmonary reanimation maneuvers obtained restoration of vital signs and sinus rhythm. The ECG after resuscitation showed non-specific abnormalities of the repolarization in the infero-lateral leads (Figure 1A). The patient had full recovery from the neurological point of view. Cardiac magnetic resonance showed late-enhancement of the left ventricular apex associated with dyskinesia in the same region and of the antero-septal portion of the same ventricle. Coronary angiography and coronary computed tomography didn't reveal any coronary artery abnormalities or critical stenosis. Echocardiographic evaluation demonstrated a left ventricular ejection fraction of 40% and confirmed the cardiac magnetic resonance findings. Final diagnosis was resuscitated cardiac arrest in a patient with left ventricular apical aneurysm of unknown origin (ischemic vs congenital). The patient finally underwent implantation of a defibrillator (ICD).

At the end of the hospitalization, an ajmaline test was performed to exclude BrS. The ajmaline test (1mg/kg of body weight) was negative for BrS, but, after 0.7 mg/kg injection (49 mg complexively), we documented an accelerated idioventricular rhythm (AIVR) with a heart rate of 95 bpm (Figure 1B). Diagnosis was confirmed after the evidence of AV dissociation and by a few fusion beats (Figure 1B and 1C). AIVR morphology was right bundle branch block (RBBB) and left anterior hemiblock (LAH). AIVR was preceded by a slight prolongation of the PQ interval (from 150 to 190 ms) and the QRS interval (from 95 ms to 130 ms). The arrhythmia ended spontaneously after termination of ajmaline infusion.

## Discussion

To the best of our knowledge, our case report is the first to describe an AIVR during ajmaline test in a patient with a resuscitated cardiac arrest and with a left ventricular apical aneurysm.

AIVR is defined as 3 or more successive wide QRS beats at a rate close to the baseline rhythm, with a maximal difference of 10-15% and with a heart rate between 100 and 120 bpm. AIVR is found to be the most common reperfusion arrhythmia in pre-thrombolytic and thrombolytic era: it is usually well tolerated [6-8] and no specific antiarrhythmic treatment is recommended apart from removing the suspected cause [9]. AIVR is believed to result from abnormal automaticity of the subendocardial Purkinje fibers owing to the washout phenomenon during infarct-related artery reperfusion [10]. Other investigators suggested triggered activity based on delayed afterdepolarizations as the underlying mechanism [11]. No association with ajmaline infusion has been described until now in the literature.

Ajmaline test is a well-recognized diagnostic tool used to unmask the electrocardiographic pattern of BrS in patients with resuscitated cardiac arrest or syncopes and in genetic carriers belonging to families affected by this pathology [12]. Among the class I blockers, ajmaline is considered the most powerful to reveal the electrocardiographic alterations typical of BrS. Among the others, propafenone has been demonstrated to be a weaker agent, probably due to the associated block of I_to_ channels. Both therapeutic, toxic and proarrhythmic effects of all sodium channel blockers are manifested on the ECG by a different degree of PR and QRS prolongation [13]. One large study using ajmaline to diagnose BrS [14] revealed a very low incidence of ventricular tachycardias (VT), around 1.3 %. All episodes of VT documented in this study were polymorphic, degenerated in ventricular fibrillation and occurred in patients in whom drug administration after the appearance of diagnostic Brugada-type ECG changes was not discontinued.

Our case substantially differs from the latter for different reasons: 1) our patient had a negative ajmaline test; 2) echocardiography showed left apical ventricular aneurysm and 3) the arrhythmia related to the intravenous administration of ajmaline was an AIVR and not a VT. Triggered activity could be addressed as potential electrophysiological mechanism leading to the cited arrhythmia, seen the capability of ajmaline of inducing early afterdepolarizations in the Purkinje fibers [15]. Another hypothesis which could explain the AIVR is macro-reentry. Indeed, class I antiarrhythmic drugs have demonstrated to potentially cause monomorphic tachycardia linked to a shortening of activation wavelength below the physical length of a preformed reentry circuit [16]. We can hypothesize an equal contribution of this two mechanisms in the physio-pathology of the cited arrhythmia in our patient. We think in fact that pathological findings evidenced by the echocardiography and the magnetic resonance together with the prolongation of conduction associated with the block of Na channels could favor a slow reentry between the left anterior and the left posterior fascicle, determining the morphology of RBBB and LAH. We would exclude a toxic effect of the ajmaline, since the AIVR appeared already before reaching the maximal usual dose of 1 mg/kg, given to elicit BrS ECG pattern.

Ajmaline-related AIVR has never been reported. In this case report AIVR was probably favored by the presence of a left ventricular apical aneurysm. Our case report postulates that ajmaline induced AIVR increasing triggered activity of Purkinje fibers or decreasing the conduction on the two fascicles of the left bundle branch, determining a slow macro-reentry in the left bundle branch. Further investigation is needed to define the mechanism underlying this finding.

## Figures and Tables

**Figure 1A:**
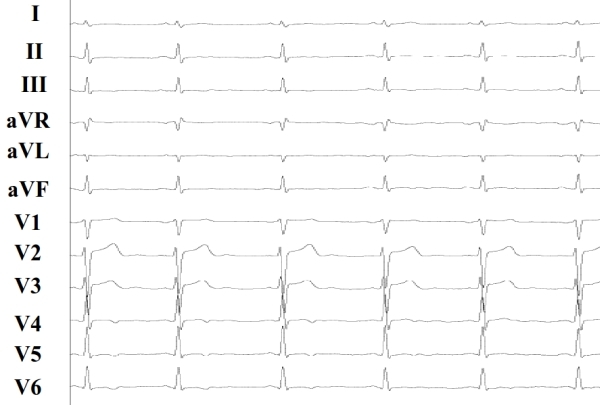
Baseline ECG showing aspecific repolarization abnormalities in the infero-lateral leads.

**Figure 1B:**
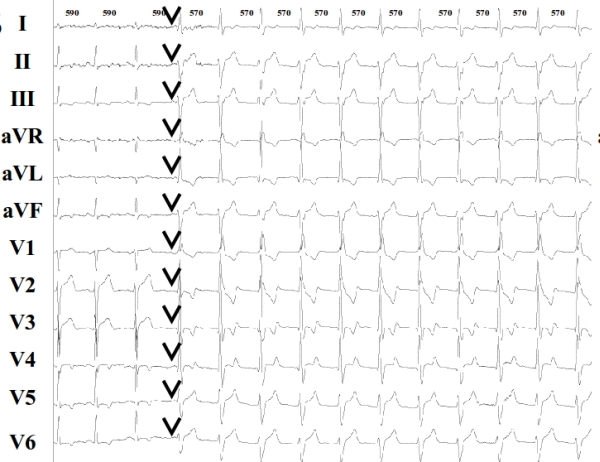
ECG showing the beginning of one episode of accelerated idioventricular rhythm (AIVR). Black arrows show P waves and isorhythmic AV dissociation. RR intervals shown on the top of the figure are expressed in ms.

**Figure 1C:**
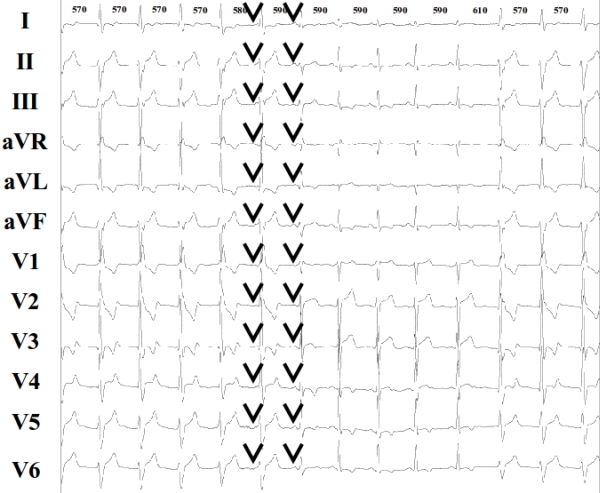
ECG showing consecutively the end of the previous episode of AIVR and the beginning of another one. Black arrows show P waves and isorhythmic AV dissociation. RR intervals shown on the top of the figure are expressed in ms.
